# Diagnostic Testing for Sepsis: A Systematic Review of Economic Evaluations

**DOI:** 10.3390/antibiotics11010027

**Published:** 2021-12-27

**Authors:** Paula Rojas-Garcia, Simon van der Pol, Antoinette D. I. van Asselt, Maarten J. Postma, Roberto Rodríguez-Ibeas, Carmelo A. Juárez-Castelló, Marino González, Fernando Antoñanzas

**Affiliations:** 1 Department of Economics and Business, University of La Rioja, 26004 Logroño, Spain; roberto.rodriguez@unirioja.es (R.R.-I.); carmelo.juarez@unirioja.es (C.A.J.-C.); marino-jose.gonzalez@unirioja.es (M.G.); fernando.antonanzas@unirioja.es (F.A.); 2 Department of Health Sciences, University of Groningen, University Medical Center Groningen, 9713 GZ, P.O. Box 30.001 Groningen, The Netherlands; s.van.der.pol@rug.nl (S.v.d.P.); a.d.i.van.asselt@umcg.nl (A.D.I.v.A.); m.j.postma@rug.nl (M.J.P.); 3 Department of Epidemiology, University of Groningen, University Medical Center Groningen, 9713 GZ, P.O. Box 30.001 Groningen, The Netherlands; 4 Department of Economics, Econometrics and Finance, University of Groningen, 9747 AE Groningen, The Netherlands

**Keywords:** sepsis, antibiotics, diagnostic testing, AMR, systematic review

## Abstract

Introduction: Sepsis is a serious and expensive healthcare problem, when caused by a multidrug-resistant (MDR) bacteria mortality and costs increase. A reduction in the time until the start of treatment improves clinical results. The objective is to perform a systematic review of economic evaluations to analyze the cost-effectiveness of diagnostic methods in sepsis and to draw lessons on the methods used to incorporate antimicrobial resistance (AMR) in these studies. Material and Methods: the Preferred Reporting Items for Systematic Reviews and Meta-Analyses (PRISMA) guidelines were followed, and the Consolidated Health Economic Evaluation Reporting standards (CHEERS) checklist was used to extract the information from the texts. Results: A total of 16 articles were found. A decision model was performed in 14. We found two ways to handle resistance while modelling: the test could identify infections caused by a resistant pathogen or resistance-related inputs, or outcomes were included (the incidence of AMR in sepsis patients, antibiotic use, and infection caused by resistant bacterial pathogens). Conclusion: Using a diagnostic technique to detect sepsis early on is more cost-effective than standard care. Setting a direct relationship between the implementation of a testing strategy and the reduction of AMR cases, we made several assumptions about the efficacy of antibiotics and the length-of-stay of patients.

## 1. Introduction

Sepsis is a life-threatening organ dysfunction caused by a dysregulated host response to infection [1]. In the period between 1990 and 2017, the number of deaths reached 11 million out of an estimated total of 48.9 million sepsis cases worldwide [2]. Although globally, general incidence and mortality have decreased by 37% and 52.8%, respectively, in this period, sepsis continues to be one of the main causes of health loss, accounting for 19.7% of deaths in 2017 [2]. A recent study concluded that the average hospital stay costs for a patient with sepsis were estimated to be EUR 11,400 [3]. Sepsis is a very serious and expensive healthcare problem that was estimated to account for 5.2% of the total cost of U.S. hospital care in 2011 [4]. Furthermore, sepsis caused by a multidrug-resistant (MDR) bacteria increases mortality and costs [5,6]. Nearly half of sepsis deaths occur following post-surgical complications or as a result of chronic illness [2]. It is especially important to note that half of all sepsis deaths occur in children [2]. The World Health Organization (WHO) has recommended including the prevention, diagnosis, and adequate antimicrobial treatment of sepsis in the strengthening of health services, as well as continuing efforts to reduce related antimicrobial resistance (AMR) [7].

To reduce the severity of sepsis complications, as well as mortality, it is essential to diagnose and initiate treatment as soon as possible after a patient’s consultation. It has been estimated that each hour of delay in starting antimicrobial treatment in patients with septic shock decreases survival by 7.6% [8]. Increased mortality has also been reported with delayed initiation of treatment in both patients with sepsis and septic shock, regardless of the number of organs affected [9]. A systematic review on the impact of the timing of antibiotic administration in severe sepsis and septic shock showed that there is no difference in mortality if the delay in starting treatment is between 1 and 5 h [10]. Moreover, another recently published systematic review indicates the importance of rapid administration of antibiotics in cases of septic shock, but it also emphasizes that this evidence is derived only from observational studies and not from randomized clinical trials [11]. It is important to target antibiotic prophylaxis in each individual patient to prevent sepsis [12]. In this sense, a study that analyzed the change in health care-associated infection in a surgical ward concluded that after an adoption of bundles of antibiotic therapy, the proportion of surgical patients receiving antibiotics dropped from 100% to 69% [13].

Analysis of blood cultures in the laboratory is currently the method used to diagnose the causative agent of sepsis with the highest certainty [1]. However, empirical antimicrobial treatment is commonly initiated before culture results are available, as these are obtained two days after collecting samples [14]. The performance of molecular microbiological diagnosis could reduce this time to a few hours [15]. Biomarkers may be useful to differentiate between patients with sepsis and those with a systemic inflammatory response not generated by infection [1]. To date, the use of biomarkers in the stage prior to the initiation of antibiotic therapy has not demonstrated sufficient diagnostic certainty to replace the performance of blood cultures [16]. However, in the interest of generating faster, more accurate, and more effective diagnostic options, biomarkers may be relevant. Several studies have evaluated the usefulness of these biomarkers for the detection of sepsis. It was concluded that procalcitonin (PCT) is the biomarker most frequently described in different guidelines due to their acceptable diagnostic performance in detecting sepsis [17]. If sepsis is ruled out, antibiotic treatment can be discontinued. Since the use of biomarkers generally requires facilities in the health services’ central laboratories, the development of point-of-care (POC) diagnostic options has attracted particular attention [1].

Another opportunity for improved diagnosis is the inclusion of the early recognition of AMR in the treatment of sepsis. Due to the multiple causes of sepsis, it is unlikely that a single biomarker is sufficient to make a diagnosis [1]. Therefore, a combination of biomarkers is the most appropriate guideline to establish a diagnosis with greater accuracy [1]. AMR in sepsis affects adult and pediatric patients. In adults, fluoroquinolone resistance in *Escherichia coli* infections has been reported to be associated with increased sepsis hospitalization rates in adults older than 50 and with higher mortality rates in adults aged 18–84 years [18].

Regarding neonates and children, there is growing concern globally about the increase in morbidity and mortality from severe infections due to AMR [19]. A review of the available evidence, especially for low- and low–middle-income countries, indicates a high incidence of AMR in neonatal sepsis [20]. It has been reported that one-third of neonatal deaths associated with sepsis are potentially related to AMR [21]. This is why it has been highlighted that AMR is already a very significant issue in neonatal care units on a global scale [21]. A further complicating aspect is the increasing global incidence of severe infections associated with MDR bacteria [18]. It has been reported that half of the pathogens causing neonatal bacterial infections are resistant to first-line (ampicillin or penicillin, and gentamicin) and second-line (third generation cephalosporins) treatments recommended by the WHO [19].

Current trends for improving sepsis diagnosis encompass at least four areas: developing new tests that increase diagnostic certainty in the period prior to the initiation of antibiotic therapy, identifying suitable biomarkers to prove infection after initiation of antimicrobial therapy, developing more effective biomarkers for use at the POC, and developing molecular bacteriological diagnostics that are more rapid than current blood cultures. In a growing number of countries, the incorporation of innovations into the standard of care requires economic evaluations to find the most cost-effective alternatives. It is also very important to consider AMR in the economic evaluation.

This paper conducts a systematic review of the economic evaluation of sepsis diagnostic methods, with specific attention for AMR. To date, no such systematic review has been published. This review may be useful in improving the current standard of care and, consequently, possibly favoring the adoption of innovative diagnostics that promote the early detection of AMR to improve treatment outcomes. The aim of this study was to conduct a qualitative synthesis of the published literature. The objectives of this article are twofold. The first objective is to carry out a systematic review of economic evaluations to analyze the cost-effectiveness of diagnostic methods in sepsis. The second objective is to draw lessons on the methods with which AMR has been incorporated into the economic evaluations included in the systematic review.

## 2. Materials and Methods

### 2.1. Type of Studies

The present systematic review was performed following the Preferred Reporting Items for Systematic Review and Meta-Analysis (PRISMA) guidelines [22]. The articles included in this review compare at least two different diagnostic strategies for sepsis. We assessed the cost-effectiveness of strategies that reduce a physician’s uncertainty when it is suspected that a patient has an infection (e.g., patients with suspected sepsis), arising from the presence of symptoms common to several diseases. Given the focus of this paper, screening studies were not included. Notably, the starting point of a screening study is that the general population is supposed to be mostly healthy and there is no a priori suspicion of disease as opposed to diagnostic testing where disease is suspected [23].

### 2.2. Search Strategy and Selection Criteria

In order to retrieve economic evaluations of diagnostic strategies for sepsis, we performed a search (syntax detailed in Supplementary File S1) in Scopus, PubMed, and Web of Science. Articles published between January 2000 and December 2020 were included in order to provide updates on clinical practice. Geographical limitations were not established. A first round of title and abstract screening was performed by P.R.-G., M.G., and S.v.d.P. After removing duplicates, we selected articles according to the inclusion criteria. In the second step, full-text reports were evaluated for eligibility. In case of any discrepancy among the reviewers, another reviewer was consulted (A.D.I.v.A.). The selection criteria have been described in more detail in a previous study [24] and were followed in this review.

### 2.3. Data Extraction and Analysis

A standardized (Google) form was used for data extraction, which was based on the Consolidated Health Economic Evaluation Reporting Standards (CHEERS) checklist [25], as recommended. We also explored whether AMR was included in the models; we therefore added this as an item. Data extraction and analysis has been detailed previously [24]. Microsoft Excel (version 2102) [26] was used to manage data extraction and transform data. The reference manager Zotero (version 5.0.82) [27] was used to store the bibliography.

## 3. Results

**Figure 1:**
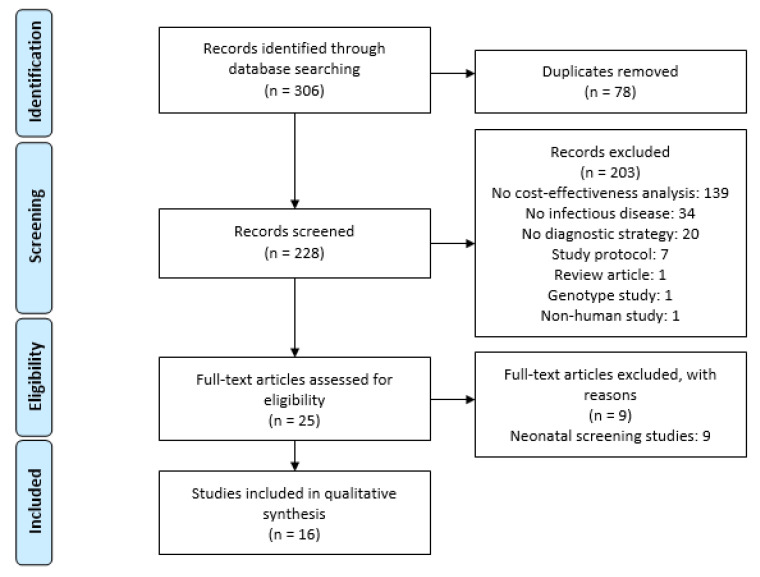
PRISMA flow diagram.

Sixteen articles studied different diagnostic strategies for sepsis. Figure 1 shows the PRISMA flow diagram of the literature review with the number of articles included and the reasons for exclusion. A total of 306 articles were found in the three peer-reviewed literature repositories. After duplications were removed (n = 78), 228 articles were screened according to their title and abstract. A total of 203 articles were excluded, mainly due to a lack of a cost-effectiveness or economic evaluation analysis (n = 139). The remaining 25 full texts were assessed, and a total of 9 were excluded because they concerned neonatal screening studies. The final result was a total of 16 cost-effectiveness articles that compared at least two different diagnostic strategies for sepsis, published between January 2000 and December 2020.

All the articles fulfilled the article descriptions items (title, abstract, objectives, target population, setting, study perspective, interventions compared, treatment, reported clinical outcomes, measurement of effectiveness, cost estimations, currency year used, type of model, assumptions taken, analytical methods, and study parameters). The items time horizon, characterizing uncertainty, source of funding, and conflicts of interest were reported in 75% of the articles (Supplementary File S2). Table 1 shows main characteristics of the papers/studies.

### 3.1. Country and Setting of the Articles

Thirteen articles performed a single-country study: the United States (USA) [32,37,39,40,41,42,43], Spain [29], Argentina [30], Italy [31], the United Kingdom [34], France [35], and the Netherlands [36]. Three articles studied more than one country: Europe and the USA [28]; Ethiopia, Gambia, Papua New Guinea, and the Philippines [33]; and the United Kingdom, Germany, and the Netherlands [38].

Articles assessed patients admitted to hospital [28,29,30,31,32,33,35,36,37,38,40,41], admitted to the intensive care unit (ICU), or presenting at the emergency department [29,32,34,36,39,41,42,43].

### 3.2. Perspective, Time Horizon, and Population

The perspectives used for the analyses were the healthcare center’s [28,29,31,32,33,34,35,36,37,39,40,41,42,43], healthcare payer’s [30], and societal perspective [38,40,41].

The modelled time horizons were the length of the hospital stay [28,30,41,42,43]; patient’s projected life expectancy [37]; or periods of 30 days [35,40], six months [34], or one year [32,36,38,39].

Three articles studied the pediatric population [30,33,34] and nine, the adult population [32,33,34,35,36,37,38,39,42]; in seven, the population was not specified [28,29,31,40,41,43].

### 3.3. Type of Model and Assessed Interventions

A decision model was performed in 14 studies [28,30,32,33,34,35,36,37,38,39,40,41,42,43]. One article performed an individual sampling model [29], and another one an observational propensity score-matched analysis [31]

Models compared standard care (broad-spectrum antibiotic treatment discontinued after a negative blood culture) with a procalcitonin (PCT) [32,34,36,38,39,41,43] or polymerase chain reaction (PCR) test [28], blood culture combined with diagnostic strategy testing (as molecular assay [42], additional testing with LightCycler^®^ SeptiFast [35] or initial specimen diversion device [40]), the Rochester criteria (a clinical scale that defines the severity of sepsis and therefore the treatment to be received) with a PCT and a PCR test [30], and clinical assessment with an unspecified point-of-care test (POCT) for sepsis [33].

One article presented an individual sampling model [29] in which patients were studied individually in two different phases. In the first phase, physicians were not aware of the result of the test, and in the second phase, they were, allowing antibiotic treatment to be adjusted in the first few hours after testing. Another article implemented a propensity score analysis, in which a retrospective group based on standard care was matched to a prospective group with an additional molecular test [31].

The vast majority of the modelling studies were based on retrospective data [29,30,31,34,39,40,41,42]. Only one study [31] analyzed prospective data in a cohort in which the PCR-based assay (SeptiFast Test) was used. In two studies, clinical trials were performed [35,36]. In the first case, this was done to compare standard care with molecular tests in blood [35], while in the second, this was done to compare with PCT [36]. Both clinical trials included patients over 18 years of age.

### 3.4. Cost-Effectiveness Results

Several outcome measures were used in the analyses: costs per quality-adjusted life-year (QALY) [32,34,36,37,39], average savings per patient [29,35,41], costs per life year [28,33], savings in hospital [38,40], average savings per case avoided [31], costs per additional correct diagnosis [30], and costs per death averted [42,43].

In five studies, the use of PCT dominated standard care as it was more efficient and less costly. In three articles, the testing strategy was cost-saving compared to standard care. Another cost-effectiveness ratio for rapid PCR testing was cost per life-year, which resulted in a cost-effectiveness ratio of USD 820 per life year in the USA and EUR 636 per life year [28]. Furthermore, it was concluded that PCR testing would be less costly than standard care even at higher prices [28]. Moreover, the testing strategy resulted in USD 147 per life saved compared to the clinical assessment strategy [33].

Two studies estimated the hospital cost savings of using a PCT strategy compared to standard care, and it was concluded that rapid testing led to annual savings in a hospital of USD 1.9 million and prevented 34 hospital-acquired conditions [40]. Moreover, total hospital costs of care per patient decreased with up to EUR 1163 [38]. In another paper, average savings per episode of EUR 430 were estimated when using a PCR [31]. Furthermore, the costs per additional diagnosis using a diagnostic test compared to using the Rochester criteria were calculated, which resulted in USD 937 per correctly diagnosed case [44]. Two papers concluded that the use of molecular tests was cost-effective, with ratios of USD 3000 [42] and USD 20,000 [43] per avoided death, respectively.

### 3.5. Antimicrobial Resistance in the Model

Antimicrobial resistance was included in nine models [28,29,32,35,38,40,41,42,43]. We found two ways to handle resistance while modelling: the test could identify infections caused by a resistant pathogen or resistance-related inputs or outcomes were included (the incidence of AMR in sepsis patients [38], antibiotic use [40] and infection caused by a resistant bacterial pathogens [41]).

Several models [28,29,32,35,42,43] considered that the test could differentiate between infections caused by resistant pathogens in the diagnostic testing strategy. Patients received treatment based on the test result, and therefore the administered antibiotic could be tailored to the patient. It was reported that a slight time delay was preferable over immediate broad-spectrum antibiotic treatment, unless clinical outcomes worsened due to this waiting time [28,35]. Moreover, with more widespread AMR, testing becomes preferable because it narrows and/or lowers antibiotic consumption [28,29,42,43]. For instance, Zachariodakis et al. [43] reported that the effectiveness of the diagnostic testing strategy is expected to maximize in hospital settings, where the prevalence of drug resistant infection is higher. Furthermore, Shehadeh et al. [42] concluded that the cost-effectiveness arises from identifying antimicrobial genes that change the initial treatment.

In order to prove testing to be preferable over immediate broad spectrum antibiotic treatment, an important assumption had to be made, i.e., that a reduction in antibiotics would immediately have an effect, lowering ICU admissions, hospital stays, and admission rates, which implies a theoretical decrease of resistant infection cases [29,32,42,43]. In Alvarez et al. [29], the testing strategy significantly reduced the number of antibiotics used in comparison to the standard care strategy (one type of antibiotic safely avoided), making the ICU length-of-stay eight days shorter in the testing strategy. In the study by Shehadeh et al. [42], the testing strategy reduced inappropriate treatment by 80% of initial inappropriate treatments, which led to a four-day reduction in hospital stays in comparison with standard care patients. With similar reasoning, in the study by Zacharioudakis et al. [43], the testing strategy reduced the length of stay by four days. It was assumed that all of these reductions of length-of-stay were due to an increase in the clinical efficacy of antibiotics due to more tailored prescriptions, which lead to a reduction of AMR.

Another assumption made was that sepsis infection was only caused by bacterial pathogens and that it is not detected in combination with other pathogens [28,29,32]. However, in one article [35], this assumption was not considered. As a result of this article that assumed that a bacterial pathogen was presented with other pathogens, no significant differences were found in the cost per patient between testing and standard care strategies because the cost was similar in both phases, as it was necessary to combat all possible co-infections. However, the testing strategy was preferred because it reduced the time between when the patient arrived and the diagnosis and treatment, leading to better clinical outcomes. One assumption made in all articles was that all physicians followed the test result and implemented the established treatment.

Steuten et al. [38] considered the incidence of AMR in sepsis patients, taking rates from the European Antimicrobial Resistance Surveillance Network (EARS-Net). They linked the duration of antibiotic treatment for sepsis therapy (in days) with the incidence of antibiotic-resistant infections. Outcomes were expressed in terms of resistant cases avoided and their costs per patient. The authors took previously published data that established that the probability of an ICU patient developing antibiotic resistance reduced by 74% for each day in which antibiotic therapy was shortened in sepsis [45,46]. The authors concluded that testing was cost-saving compared to standard care and led to a reduction of days in ICU, which implies a significant number of resistant infection cases avoided. An assumption of this model was that infections caused by a resistant pathogen solely occurred in the ICU.

Two articles [40,41] considered resistance-related outputs in the model. Antibiotic use and hospital-acquired conditions were reported outcomes of one model [40]. The authors assumed that these outcomes were likely to generate adverse clinical consequences, such as the development of a resistant infection. However, most of the input data were taken from the literature or from expert opinion. The testing strategy led to lower costs and adverse clinical events in this case.

Mewes et al. [41] reported resistant infection cases detected as an outcome. In addition, antibiotic days safely avoided were included. With a prevalence of 21.7% of AMR infections related to sepsis in the United States, it was considered that one antibiotic day safely avoided led to a 3.2% reduction of total AMR cases [45,46,47]. This number was multiplied by the difference in antibiotic days between the testing strategy and standard care. The testing strategy was cost saving and a reduction of 16,000 resistant infection cases was estimated in the whole U.S. population in one year. Moreover, assumptions were made regarding test and hospital stay costs.

Articles that did not include AMR in the model recognized this as a limitation of their study [31,33,34,36,37,39].

Two articles [31,33] mentioned that a diagnostic testing strategy, detecting resistant infections, could have been included in the model. The authors presumed that this may result in long-term savings, since resistant infections generate a higher cost than non-resistant infections. However, the authors stated that a diagnostic testing strategy was not included because the study was not designed for this purpose [31], such tests have not been developed in low-resource settings [33], and the current protocols do not include the implementation of a resistant infection test or the development of a database to facilitate the administration of these patients [33].

Five articles considered the fact that they excluded the impact of the reduction of antibiotics on the prevalence of AMR from their analysis to be a limitation. They believed that the benefits of the diagnostic test strategy may have been underestimated because of this. However, the authors claimed that it was difficult to account for all possible adverse events from antibiotic use and it could bias the results towards the testing strategy [34,37,39], the literature found on correctly quantifying this impact was limited [32,34,36], performing a long-term analysis was challenging, and future effects of the reduction of antibiotics in the resistant infection cases were difficult to quantify [34].

## 4. Discussion

In this literature review, 16 articles that compared different diagnostic strategies for sepsis were retrieved and analyzed following the PRISMA guidelines [22]. In general, the articles included most items from the CHEERS checklist [25]. Analyses mainly used a decision tree model to compare standard care, usually consisting of a blood culture and initial empiric treatment with broad-spectrum antibiotics, to performing a PCR test with more tailored treatment. Costs were assessed from a healthcare center’s perspective most often. Diagnostic testing was the preferred strategy in all articles. The clinical and economic benefits of both alternatives were equivalent when sepsis was assumed to be produced by a combination of pathogens, rather than a bacterial source only.

In the present literature review, the time horizon was mainly short, usually less than one year, although sepsis can have long-term effects on mortality and quality of life [48]. The only model with a projected life expectancy time horizon reported the information on mortality for only the first 30 days after patient admission. Analyses were not able to explore long-term costs and effects beyond two years.

Some of the reviewed papers were nearly cost analysis studies and did not calculate the ICERs [29,35,41]. It is common in many publications that apparently perform economic evaluation only to calculate cost per case and savings and not establish a comparison versus the other alternatives. Nevertheless, we included these studies to provide a wider view of the economic studies related to the sepsis diagnostic.

Incorporating the effects of AMR into the model (for instance, increasing the days of hospitalization) was reported to be difficult because the authors could not quantify this. As it is necessary to consider AMR in patients with sepsis to establish adequate treatment and reduce mortality [49], we paid special attention to how the models handled AMR. This phenomenon was included in nine articles, with two different considerations: (1) the authors assumed that diagnostic tests enabled targeted antibiotic treatment, allowing broad-spectrum antibiotics to be safely avoided; (2) the authors considered the incidence of AMR in sepsis patients and related the number of days on antibiotics and hospital-acquired conditions developed to the development AMR.

The testing strategy reduces inappropriate treatment, leading to better clinical results in comparison to standard care. However, to establish a direct relation between the implementation of a testing strategy and the reduction of AMR cases, an assumption is made: the clinical efficacy of antibiotics increases because of their more rational use. This is shown in the model through a shortened hospital length of stay. It was found that direct effects between the testing strategy, which saves the use of one type of antibiotic [29] or reduces the broad-spectrum treatment in 80% of patients [42,43], leads to a reduction of 4 to 8 days in hospital stay, and therefore the clinical efficacy of antibiotics is increased. Furthermore, it was considered that one antibiotic day safely avoided resulted in a reduction of 3.2% of AMR [41], and the probability of a particular patient developing antibiotic resistance is reduced by 74% [38]. However, it was acknowledged that AMR is influenced by other factors such as the veterinary field and food industry [50,51], therefore making it difficult to directly link antibiotic reduction to a corresponding AMR reduction.

As several ratios are used to show the cost-effectiveness of each intervention, it is not possible to compare them and decide the most cost-effective strategy across all the reviewed studies. In that sense, it would have been desirable the use of QALYs to allow comparisons between interventions. Further, the result of the estimations of QALYs gains, as well as the cost per patient, per hospital, and per avoided death make the use of rapid diagnostic test a useful tool for obtaining significant savings and improved health outcomes.

As mentioned in the introduction, the aim of this study was to conduct a qualitative syntax as it is more feasible to take into account the features of the cost-effectiveness studies. We have to bear in mind that according to Mastrigt et al. [52], the results of economic evaluations are difficult to synthesis in a quantitative way, as “there are currently no agreed-upon methods for pooling combined estimates of cost-effectiveness (e.g., incremental cost-effectiveness, cost–utility, or cost–benefit ratios), extracted from multiple economic evaluations, using meta-analysis or other quantitative synthesis methods”.

New tests for sepsis are being developed, but they are currently only in the research stages [16]. Another assumption that highlights the slow development of diagnostic tests for sepsis was that the test’s sensitivity and specificity rates were based on the authors’ assumptions. For a new diagnostic test to be approved, it is recommended that an improvement in performance compared to other diagnostic options such as cultures be evidenced [16]. Taking into account the present literature review, it was found that a preferable diagnostic strategy for sepsis should (1) report the time to correct diagnosis as an outcome, as clinical benefits and cost between alternatives could be similar due to high costs and complications; (2) include AMR in the analysis by considering the use of a test that targets resistant infections or by using AMR-related inputs in the model; and (3) extend the time horizon of the analysis in order to capture possible future adverse events and long-term sequelae in sepsis patients.

Two studies remarked the necessity of identifying sepsis as a fundamental factor to improve survival rates [53,54]. Furthermore, one of the studies highlighted an additional cost item derived by sepsis, that is, the litigation costs related to medico-legal and insurance issues [53]. However, none of the studies in our review included this type of cost.

Comparison of the impact of diagnostic alternatives on the standard of care can benefit greatly from the implementation of clinical trials. The use of strict criteria for comparing groups in clinical trials, as well as randomization, are significant strengths [55]. In recent years, adaptive designs have been proposed in clinical trials with the purpose of increasing flexibility for their execution [56,57]. These innovative designs have also been highlighted for the clinical development of antibacterials [58].

The need for implementation of clinical trials to compare diagnostic alternatives for sepsis in children was emphasized [55] due to the magnitude of sepsis mortality in this group. The fact that papers identified in this systematic review included mainly adult populations clearly demonstrates the priority of generating evidence to reduce the impact of sepsis in the pediatric group. The right of children to have the same quality of information obtained through clinical studies as adults has also been highlighted recently [59]. This knowledge gap regarding the treatment of children has been recognized in legislation passed in North America and the European Union [60].

## 5. Conclusions

Using a diagnostic technique to detect sepsis early is cost-effective compared to standard care. However, diagnostic techniques for sepsis are currently being developed, and meanwhile it is inevitable to apply several assumptions in performing economic evaluations. Given the short time horizons of sepsis episodes, simple decision tree schemes are usually applied in the models to assess the value of diagnostic techniques. The link between the development of AMR and use of antibiotics—including the spectrum and treatment duration—is difficult to quantify. Therefore, different scenarios must be defined to simulate the efficiency of these new diagnostic instruments.

There are different techniques that have been compared to diagnose sepsis. According to the present review, PCR and PCT are more efficient than standard care, as QALY gains have been found together with savings for the health system. In this sense, the review in terms of efficiency confirms what has been stated in some clinical guides.

## Figures and Tables

**Table 1:** Sepsis diagnostic articles.

First Author (Year)	Country	Setting	Perspective, Time Horizon, and Population	Type of Model	Strategies Compared (*)	Cost-Effectiveness Results (*)	Turn-Around	Treatment	AMR Included	Uncertainty Reported
Brown (2010) [28]	Europe and USA	Hospital	Healthcare center’s - The length of the hospital stay - Unspecified	Decision tree	(1) Empiric vancomycin; (2) semi-synthetic penicillin; **(3) PCR that distinguishes MRSA and MSSA**	In EU (1) EUR 695 per life-year saved; (2) EUR 687 per life-year saved; **(3) EUR 636 per life-year saved**. In USA (1) USD 898 per life-year saved; (2) NA; **(3) USD 820 per life-year saved**	(3) In less than 1 h	Semi-synthetic penicillin if MSSA and vancomycin if MRSA	Test can detect and differentiate between MSSA and MRSA (treatment is guided)	DSA, sensitivity analysis graph
Alvarez (2012) [29]	Spain	Hospital and ICU	- Healthcare center’s - 6 months - Unspecified	Individual sampling model	**(1) PCR**; (2) standard care (broad-spectrum antibiotic)	**(1) EUR 32,228 per patient**; (2) EUR 42,198 per patient	(1) In a few hours	Antibiotic treatment	Test can narrow the spectrum of antibiotics and lower rate of ICU patients	DSA
Buendía (2013) [30]	Argentina	Hospital	- Healthcare payer’s - The length of the hospital stay - Pediatric	Decision tree	(1) PCT; **(2) PCR**; (3) Rochester criteria	(1) USD 943 per correctly diagnosed case; **(2) USD 937 per correctly diagnosed case**; (3) USD 1241 per correctly diagnosed case	NA	Antibiotic treatment	No	DSA, tornado diagram
Mancini (2014) [31]	Italy	Hospital	- Healthcare center’s - 2 years - Unspecified	Observational, propensity score-matched analysis	**(1) PCR**; (2) standard diagnostic assays	**(1) EUR 1579 per patient**; (2) EUR 2010 per patient	NA	Antibiotic treatment	Mentioned as a limitation	PSA
Harrison (2015) [32]	USA	Hospital and ICU	- Healthcare center’s - 1 year - Adults	Decision tree	**(1) PCT**; (2) standard care (broad-spectrum antibiotic)	**(1)** vs. (2) +0.0002 QALYs gained and − USD 65 per patient USD 245,501 (ICER)	NA	Vancomycin and cefepime	Test can detect and differentiate between MSSA and MRSA (treatment is guided)	DSA, PSA
Penno (2015) [33]	Ethiopia, Gambia, Papua New Guinea, and the Philippines	Hospital	- Healthcare center’s - NA - Adults and pediatric	Decision tree	**(1) POCT**; (2) clinical assessment	**(1)** vs. (2) + USD 147 per life saved (lowest prevalence) **(1)** vs. (2) + USD 4988 per life saved (highest prevalence)	(1) Results available in a timeframe that can inform initial patient management.	Ampicillin, gentamicin, and ceftriaxone	Mentioned as a limitation	DSA, sensitivity analysis graph
Westwood (2015) [34]	United Kingdom	ED and ICU	- Healthcare center’s - 6 months - Adults and pediatric	Decision tree	**(1) PCT**; (2) standard care (broad-spectrum antibiotic)	**(1)** vs. (2) +0.005 QALYs gained	NA	Antibiotic treatment	Mentioned as a limitation	DSA, PSA, CE plane, CE acceptability curve
Cambau (2017) [35]	France	Hospital	- Healthcare center’s - 30 days - Adults	Decision tree	(1) Blood cultures; **(2) LSF**	**(2)** vs. (1) − EUR 535 per patient	(1) 2–3 days (2) a shorter time to results	Beta-lactams, cephalosporins, and other antibiotics	Test can detect resistant infection (treatment is guided)	PSA, CE plane
Kip (2018) [36]	The Netherlands	Hospital and ICU	- Healthcare center’s - 1 year - Adults	Decision tree	**(1) PCT**; (2) standard care (broad-spectrum antibiotic)	**(1) EUR 46,081 and +0.47 QALY per patient gained** (2) EUR 46,146 per patient	(1) Result available in the first 24 h	Antibiotic treatment	Mentioned as a limitation	CE plane, CE acceptability curve
Pliakos (2018) [37]	USA	Hospital	- Healthcare center’s - Projected life expectancy of the patients (death considered only in the first 30 days after admission) - Adults	Decision tree	12 strategies: **MALDI-TOF analysis with an ASP**; conventional laboratory methods without an ASP; others	Rapid diagnostic tests results in less than 24 h. MALDI-TOF resulted in + USD 29,205 per quality-adjusted life year compared to conventional laboratory methods	Conventional laboratory methods up to 5 days for Results.	Antibiotic treatment	Mentioned as a limitation	CE plane, CE acceptability curve, PSA
Steuten (2018) [38]	United Kingdom, Germany, and the Netherlands	Hospital	- Societal - 1 year - Adults	Decision tree	**(1) PCT**; (2) standard care (broad-spectrum antibiotic)	**(1)** vs. (2) − EUR 1071 (Germany), – EUR 1124 (the Netherlands), and −EUR 1163 (UK) hospital costs. Societal cost savings of + EUR 1309; + EUR 1371, and + EUR 1321 per patient, respectively.	NA	Antibiotic treatment based on the concentration of PCT	The incidence of AMR was included in the model	DSA, tornado diagram, sensitivity analysis graph, PSA
Collins (2019) [39]	USA	ICU	- Healthcare center’s - 1 year - Adults	Decision tree	**(1) PCT**; (2) standard care (broad-spectrum antibiotic)	**(1)** vs. (2) +0.0001 QALYs gained and -USD 45 per patient	(1) Result available in the first 24 h	Antibiotic treatment	Mentioned as a limitation	PSA
Geisler (2019) [40]	USA	Hospital	- Societal and healthcare center’s - 30 days - Unspecified	Decision tree	(1) Blood cultures; **(2) ISDD**; (3) phlebotomists	**(2)** annual savings in a hospital of USD 1.9 million and prevent 34 hospital-acquired conditions	NA	Antibiotic treatment	Antibiotic use and adverse clinical consequences as outcomes of the model	DSA, tornado diagram
Mewes (2019) [41]	USA	Hospital and ICU	- Societal and healthcare center’s - The length of the hospital stays - Unspecified	Decision tree	**(1) PCT**; (2) standard care (broad-spectrum antibiotic)	**(1)** vs. (2) saved USD 11,311 per patient	(1) Result available in the first 24 h	Antibiotic treatment	Patients with antibiotic resistant infections and antibiotics as outcomes of the model	DSA, tornado diagram of DSA
Shehadeh (2019) [42]	USA	ICU	- Healthcare center’s - The length of the hospital stays - Adults	Decision tree	(1) Only blood culture; **(2) molecular testing and blood culture**	(2) vs. **(1)** USD 3000 per death averted	(2) In 2–7 h	Antibiotic treatment	Test can detect resistant infection (treatment is guided)	DSA
Zacharioudakis (2019) [43]	USA	ED	- Healthcare center’s - The length of the hospital stays - Unspecified	Decision tree	**(1) PCT**; (2) standard care	**(1)** vs. (2) − USD 20,000 per death averted	NA	Antibiotic treatment	Test can detect resistant infection (treatment is guided)	DSA

^(^*^)^ the strategies that were considered to be cost-effective by the authors are in bold. ASP (antimicrobial stewardship program); CE (cost-effectiveness); DSA (deterministic sensitivity analysis); ED (emergency department); HACs (hospital-acquired conditions); ICER (incremental cost-effectiveness ratio); ICU (intensive care unit); ISDD (initial specimen diversion device); LSC (lightCycler SeptiFast); MALDI-TOF (matrix assisted laser desorption ionization–time of flight); MRSA (methicillin-resistant Staphylococcus aureus); MSSA (methicillin-susceptible S. aureus); NA (not reported); PCR (polymerase chain reaction); PCT (procalcitonin); POCT (point-of-care test); PSA (probabilistic sensitivity analysis); QALY (quality-adjusted life-year); SBI (serious bacterial infections); SIRS-SS (systemic inflammatory response syndrome).

## Supplementary Materials

The following are available online at https://www.mdpi.com/article/10.3390/antibiotics11010027/s1. Supplementary File S1 Syntax used in the search to retrieve economic evaluation of diagnostic strategies for the management of sepsis. Supplementary File S2 CHEERS Checklist Results.
