# Supplementary material for: Diagnostic Testing for Sepsis: A Systematic Review of Economic Evaluations
Source: Antibiotics (Basel). 2021 Dec 27;11(1):27. doi: 10.3390/antibiotics11010027 (PMC8773030; doi:10.3390/antibiotics11010027)
Supplement: Supplementary file 1 [file antibiotics-11-00027-s001.zip › antibiotics-1482266-supplementary.pdf]

Supplementary File 1 Syntax used in the search to retrieve economic evaluation of diagnostic strategies for the management of sepsis.

SCOPUS

(TITLE-ABS-KEY(pharmacoeconomic *)
OR TITLE-ABS-KEY(cost-effectiveness)
OR TITLE-ABS-KEY("economic evaluation")
OR TITLE-ABS-KEY("health technology assessment"))
AND (TITLE-ABS-KEY(antibiotic*)
OR TITLE-ABS-KEY(infectious)
OR TITLE-ABS-KEY("bacterial infection")
OR TITLE-ABS-KEY("viral infection"))
AND (TITLE-ABS-KEY("diagnostic")
OR TITLE-ABS-KEY("diagnostics")
OR TITLE-ABS-KEY("test")
OR TITLE-ABS-KEY("tests")
OR TITLE-ABS-KEY("testing"))
AND (TITLE-ABS-KEY("sepsis"))
AND PUBYEAR >1999
AND PUBYEAR <2020

PUBMED

((infectious
OR "bacterial infection"
OR "viral infection"
OR antibiotic *
OR antimicrobial)
AND ("diagnostic"
OR "diagnostics"
OR "test"
OR "tests"
OR "testing")
AND (pharmacoeconomic *
OR "cost-effectiveness"
OR "economic evaluation"
OR "health technology assessment")
AND ("sepsis")
) AND (("2000/01/01"[Date - Publication] : "2020/12/31"[Date - Publication]))

WEB OF SCIENCE

TS = ((("bacterial infection"
OR "viral infection"
OR antibiotic *
OR antimicrobial
OR infectious)
AND ("diagnostics"
OR "diagnostic"
OR "test"
OR "tests"
OR "testing")
AND (pharmacoeconomic*
OR cost-effectiveness

OR “economic evaluation”
OR “health technology assessment”)
AND (“sepsis”)))
Period of time: 2000–2020

Supplementary File S2 CHEERS Checklist Results

CHEERS Item	Percentage of Articles that Reported the Item (n = 16)
Title	100%
Abstract	100%
Background and objective	100%
Target population	100%
Setting	100%
Study perspective	100%
Interventions compared	100%
Treatment	100%
Time horizon	93.7%
Discount rate for health outcomes	6.3%
Discount rate for economic outcomes	0%
Reported clinical outcomes	100%
Measurement of effectiveness	100%
Resource and cost estimations	100%
Currency year used	100%
Type of model	100%
Assumptions	100%
Analytical methods	100%
Study parameters	100%
Characterizing uncertainty	93.7%
Study findings, limitations, generalizability, and current knowledge	100%
Source of funding	87.5%
Conflicts of interest	75%
